# Supersaturated Oxygenation: Impact on Microvascular Obstruction?

**DOI:** 10.1016/j.jscai.2024.101978

**Published:** 2024-04-06

**Authors:** Timothy D. Henry, Saraschandra Vallabhajosyula, Jay H. Traverse

**Affiliations:** a The Carl and Edyth Lindner Center for Research and Education at The Christ Hospital Health Network, Cincinnati, Ohio; b Warren Alpert Medical School of Brown University and Lifespan Cardiovascular Institute, Providence, Rhode Island; c Minneapolis Heart Institute Foundation at Abbott Northwestern Hospital, University of Minnesota School of Medicine, Cardiovascular Division, Minneapolis, Minnesota

**Keywords:** acute coronary syndrome, microvascular disease, percutaneous coronary interventions, ST-segment-elevation myocardial infarction, supersaturated oxygen

Primary percutaneous coronary intervention (PCI) for the treatment of ST-segment elevation myocardial infarction (STEMI) has significantly reduced morbidity and mortality compared with thrombolytic therapy, but there continues to be considerable long-term morbidity, including the development of heart failure (HF).^1^ This is likely due, in part, to our inability to abrogate ischemia/reperfusion injury (IRI). A consequence of IRI is the development of microvascular obstruction (MVO) and intramyocardial hemorrhage that result in infarct expansion and severe microvascular injury.^2^

MVO is a consequence of both intravascular and extravascular factors, including embolization of plaque debris, vasoconstriction, microvascular dysfunction, and compressive myocardial edema.^2^, ^3^, ^4^ It is likely that both extracellular and intracellular edema contribute to compression of the microcirculation, which may manifest as MVO. The dependence of edema on IRI is reflected in its absence in the setting of coronary artery occlusion without reperfusion. MVO occurs in nearly 50% to 60% of STEMI patients and is associated with greater infarct size and adverse left ventricular (LV) remodeling resulting in higher rates of mortality and HF hospitalizations.^2^, ^3^, ^4^, ^5^ Unfortunately, no consistent therapy has been shown to reduce MVO in the setting of STEMI and primary PCI.

Intracoronary delivery of hyperoxic blood (ie, supersaturated oxygen [SSO_2_]) has been associated with decreased endothelial edema, higher capillary vasodilatation, and decreased infarct size in animal models and clinical trials.^6^, ^7^, ^8^, ^9^, ^10^ The efficacy in reducing infarct size has been attributed to the ability of SSO_2_ to achieve significantly higher levels of dissolved oxygen in plasma available for diffusion into the ischemic zone as well as a counter-intuitive reduction in harmful free radical generation. The AMIHOT (Acute Myocardial Infarction with Hyperoxemic Therapy) trial found SSO_2_ to be effective in reducing infarct size in a subgroup of patients with anterior STEMI receiving SSO_2_ within the first 6 hours of symptom onset as measured by single photon emission computed tomography imaging 2 to 3 weeks following PCI.^7^ Subsequently, the AMIHOT-II trial randomized 301 patients with anterior STEMI to 90 minutes of intracoronary SSO_2_ delivery to the left anterior descending artery via a 7F guide catheter (n = 222) compared with 79 control patients and showed comparable major acute cardiovascular outcomes and reduced infarct size.^8^ The subsequent IC-HOT study (Evaluation of Intracoronary Hyperoxemic Oxygen Therapy in Anterior Acute Myocardial Infarction) noted a reduction in net adverse clinical events including all-cause death, new-onset HF, or HF hospitalization and reduced infarct size compared with a propensity-matched subset of patients derived from the INFUSE-AMI (Intracoronary Abciximab and Aspiration Thrombectomy in patients with Large Anterior Myocardial Infarction) trial.^9^

In this issue of *JSCAI*, Falah et al^10^ evaluated the potential of SSO_2_ delivery to reduce MVO. They compared patients with anterior STEMI (not complicated by cardiogenic shock) enrolled in 2 small SSO_2_ clinical trials (Optimized SSO_2_ pilot and IC-HOT [n = 90]) to a control population of similar patients enrolled in 1 of 7 randomized trials of anterior STEMI who received various interventions but who did not receive SSO_2_ (n = 784).^10^ All patients underwent primary PCI of the proximal or mid-LAD within 6 hours of symptom onset. SSO_2_ was delivered through the left main coronary ostium for 60 minutes using a standard 5F diagnostic catheter.^11^ MVO was assessed by cardiac MRI within 10 days of PCI. The population was largely male (>80%) and middle-aged (median age, 59 years), with an average ischemic time of 2.5 hours and >95% in both groups achieving TIMI 3 flow.

In the 90 patients who received SSO_2_, a total of 48 patients (53.3%) had MVO compared with 58.5% of 784 patients from the control group (*P* = .35). SSO_2_ therapy was associated with a lower extent of MVO (0.2% of LV mass in the SSO_2_ group and 0.8% in the control group; *P* = .052). After multivariable analysis, SSO_2_ was associated with a lower extent of MVO compared with STEMI patients who did not receive SSO_2_ (*P* = .03).^10^

Previous studies have observed that the presence of MVO following STEMI has been associated with adverse LV remodeling, HF, and increased mortality in the subsequent 1 to 2 years of follow-up.^2^, ^3^, ^4^ That MVO is associated with adverse outcomes is not surprising given patients with MVO frequently have greater infarct size and LV volumes and reduced LV ejection fraction. The unanswered question is why an entity entirely contained within the infarct zone and occupying just a few percent of LV mass can have such an outsized influence on the long-term cardiovascular outcomes in patients. Thus, the finding that an intervention such as SSO_2_ that may reduce MVO and infarct size may be a powerful therapy for STEMI patients.^8^

However, such an important claim should come with a high burden of proof. That SSO_2_ therapy could reduce MVO makes clinical sense given preclinical studies demonstrating that SSO_2_ therapy reduces endothelial cell swelling and necrosis in the microcirculation.^12^ Recent clinical studies have demonstrated that anterior STEMI patients with elevated angiographic index of microcirculatory resistance (IMR) experience a significant decline in IMR following SSO_2_ therapy, consistent with improving microcirculatory function. In contrast, patients without an elevated IMR showed no reduction in IMR following SSO_2_ therapy.

We agree with the authors that their findings that SSO_2_ therapy may reduce MVO remain hypothesis-generating, especially in light of all the adjunctive therapies that patients in the control group received and the lack of comparative infarct size data between the 2 groups. Given the potential benefit of SSO_2_, why has the clinical uptake been so slow? SSO_2_ requires 60 minutes to deliver, which can be a challenge in a busy cardiac catheterization laboratory. This is further magnified during off-hours STEMI activations and the postpandemic challenge of health care worker shortages and hospital economics. These practicalities must be weighed against the potential benefit from SSO_2_ use in this population. This patient population received early and successful PCI in moderate-sized anterior STEMI; however, patients presenting late and with larger infarcts may benefit even more.

The central issue remains identification of the appropriate patient population that may benefit from SSO_2_ therapy and the urgent need to perform head-to-head comparisons with standard of care in randomized trials. Development of a risk calculator for MVO using a combination of prehospital, hospital-based, and procedural-based variables may help in identifying high-risk patients, but post-PCI invasive physiology tools, such as IMR, may further enhance this algorithm in detecting patients at the highest risk of MVO.^13^ Ultimately, identification of high-risk patients should facilitate more targeted use of SSO_2_ and other therapies designed to decrease MVO and infarct size.
